# Toxic Shock Syndrome From Group C Streptococcus

**DOI:** 10.7759/cureus.28190

**Published:** 2022-08-19

**Authors:** Jocelyn McCullough, Shehnaz Wasim, Kuschner Zachary, Philip Nizza

**Affiliations:** 1 Medical Education, Zucker School of Medicine, Port Jefferson, USA; 2 Critical Care Medicine, Zucker School Of Medicine, Port Jefferson, USA

**Keywords:** critical care, gram positive bacteremia, bullous cellulitis, intravenous immunoglobulins (ivig), toxic-shock syndrome, group c streptococcus

## Abstract

We present a patient who was admitted with lower extremity cellulitis and was found to have Group C *Streptococcus* bacteremia causing toxic shock syndrome. Our patient was started on appropriate antibiotics, which included piperacillin/tazobactam, vancomycin, and clindamycin for presumed cellulitis, and was later transitioned to meropenem on day two when she was found to have gram-positive group C bacteremia and was treated for 14 days. Additionally, she was initiated on a three-day regimen of intravenous immunoglobulin (IVIG) as an adjunctive treatment for worsening clinical status from toxic shock syndrome. Our patient survived up to 46 days post admission but ultimately succumbed to her illness. It is worthwhile to state that the addition of IVIG could have prolonged her survival. We emphasize the importance of timely diagnosis and treatment with antibiotics and IVIG to help prevent mortality from this condition.

## Introduction

Based on 1996 classification, *Streptococcus dysgalactiae* subspecies *equisimilis* (SDSE), is one of two Lansfield groupings, Groups C and G streptococci. This bacterium is a part of human flora in the upper airway, skin, gastrointestinal tract, and female genital tract. Clinically it has been associated with a wide range of skin and soft tissue infections such as impetigo, cellulitis, and necrotizing fasciitis [1-6]. It has also been associated with bacteremia, osteomyelitis, neonatal sepsis endocarditis, and in worse case scenarios streptococcal toxic shock syndrome (TSS) [7-9]. 

## Case presentation

A 93-year-old female with a history of hypertension, heart failure with reduced ejection fraction, and chronic atrial fibrillation with a pacemaker presented with symptoms of worsening lower extremity edema and erythema for two days prior to admission. On initial presentation, she was afebrile, hypotensive with a blood pressure of 68/35 mmHg, tachycardic with a heart rate of 90, tachypneic with a respiratory rate of 33 breaths per minute, and hypoxic with oxygen saturation of 86% on room air. The patient was awake but confused, pupils were equal and reactive to light, auscultation of lungs revealed scattered rales bilaterally and she had 2+ pitting edema of her lower extremities up to her knees. There was also visible erythema, warmth, and tenderness on palpation of the left lower extremity. The patient's white blood cell count was 5.92 K/Ul ( 5-10 K/Ul) and she had thrombocytopenia with platelets of 89K/Ul (150-400k/Ul). Her lactate was elevated at 4.6 mmol/L (0.5-2.2 mmol/L) and so was her pro-calcitonin at 3.95 ng/ml (<0.5ng/ml). Additionally, she was found to have elevated pro-B-type natriuretic peptide (proBNP) of 9568 pg/mL (1-450 pg/mL), and elevated troponin T of 58 ng/L (<= 14ng/L). The patient’s chest x-ray showed mild to moderate central vascular congestion. Her electrocardiogram (ECG) showed paced ventricular rhythm. She had a non-contrast computed tomography (CT) of the head, which did not show any evidence of intracranial bleeding. CT of the chest with contrast showed small bilateral pleural effusions with mild pulmonary vascular congestion, cardiomegaly, and right heart strain without any evidence of pulmonary embolism. CT scan of her lower extremities without intravenous (IV) contrast showed diffuse subcutaneous edema without any subcutaneous emphysema.

Based on her clinical presentation the patient met systemic inflammatory response (SIRS) criteria for sepsis from cellulitis. The patient was found to be hypoxic based on the partial pressure of oxygen (PO2) and was transitioned from nasal cannula to bilevel positive airway pressure. Her blood pressure did not respond well to boluses of intravenous fluids (IVF) and hence required a right internal jugular catheter placed and was started on norepinephrine, vasopressin, phenylephrine, and epinephrine pressor support to maintain a target mean arterial pressure (MAP) goal of 65-70 mm Hg. On day one of admission, she was initiated on piperacillin/tazobactam, vancomycin, and clindamycin for broad-spectrum antibiotic coverage for cellulitis. Transthoracic echocardiogram showed severely reduced left ventricular systolic function with an ejection fraction (LVEF) of 25-30% and severe global hypokinesis of the left ventricle and severe tricuspid regurgitation and severe mitral valve regurgitation. Since the patient had decreased left ventricular ejection fraction (LVEF), she was started on additional dobutamine for potential cardiogenic shock in addition to her septic shock. 

**Figure 1:**
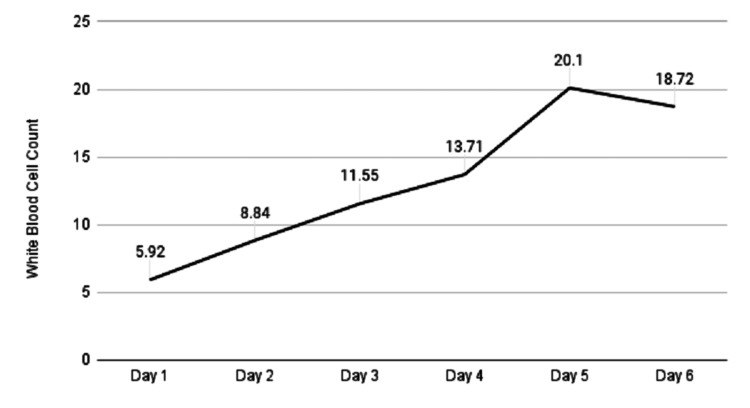
Trend in white blood cell count after initiation of IVIG on day two IVIG: intravenous immunoglobulin

On day two of admission, the patient was intubated for airway protection and placed on fentanyl and midazolam drips to maintain adequate sedation of a Richmond agitation sedation scale (RASS) goal up to -4 since the patient was breathing over the vent. Her blood cultures (both aerobic and anaerobic bottles) were positive for Group C *Streptococcus*. She was seen by infectious disease and her antibiotics were changed to meropenem, clindamycin and additionally, she was treated with IVIG (1 g/kg on day one, followed by 0.5 g/kg on days two and three) for streptococcal toxic shock syndrome, which was initiated on day two of admission (Figure 1).

The patient had worsening lower extremity edema with blister formation and hence general surgery was consulted for fascial cut-down to rule out necrotizing fasciitis. However, there was no evidence of tissue decay with fascial cut-down. Wound cultures were negative for bacterial infection. She continued meropenem for 14 days to treat bacteremia. Her stay at the ICU was complicated with a retroperitoneal bleed requiring blood transfusions. On day 29 post admission, she had a percutaneous endoscopic gastrostomy (PEG) tube and tracheostomy placed since she had failed her swallow evaluations and spontaneous breathing trials. Unfortunately, her clinical condition worsened secondary to worsening cardiogenic shock refractory to medical treatment and she died on day 46 post admission. 

## Discussion

Group A *Streptococcus* and *Staphylococcus aureus* are usually associated with TSS [7-9]. These gram-positive cocci release exotoxins, which act like superantigens, activating the immune system with a large release of cytokines triggering the inflammatory response. SDSE has virulence determinants like *Streptococcus pyogenes*, which includes streptolysin O, streptolysin S, antiphagocytic M protein, and exotoxins causing TSS [10]. This condition usually presents with signs of infection associated with skin irritation, blisters, and signs of cellulitis. Patients can very rapidly develop signs of sepsis with hemodynamic compromise leading to multiorgan failure and in worst-case scenarios, fatal death. TSS from SDSE has a very high mortality rate of 15%. The incidence of group C/G bloodstream infection was estimated at 5.5 per 100,000 population in a population-based surveillance in British Columbia, Canada, from 2011 to 2018, which identified 210 episodes of bloodstream infection due to beta-hemolytic streptococci [11]. 

Both group C and group G *Streptococcus* are susceptible to beta lactams such as penicillin G, third-generation cephalosporins, and for those who are hypersensitive, vancomycin, macrolides, and clindamycin. The duration of treatment with antibiotics depends on the type of infection, seven to 10 days for cellulitis, 14 days for bacteremia, 14-28 days for severe invasive soft tissue infection, 28-42 days for osteomyelitis, septic arthritis, and endocarditis. 

The use of IVIG in toxic shock syndrome has been shown to increase the plasma neutralizing activity of superantigens and, hence, reduce T cell production of interleukin-6 and tumor necrosis factor-alpha [12]. In a European randomized double-blinded, placebo-controlled trial that took place in 1999 regarding the use of IVIG in patients with streptococcal TSS, 120 patients were randomly assigned to receive IVIG vs placebo [13]. The trial was prematurely terminated due to slow patient recruitment and data was available for only 21 patients (11 patients received IVIG and 10 patients received placebo). There was a higher 28-day mortality of 3.6-fold in the placebo group compared to the IVIG group, though statistical significance wasn’t reached due to the small sample size. However, there was clinical significance in patients receiving IVIG, which was evident from a decrease in the sequential organ failure assessment (SOFA) score, which is used to assess the incidence of organ dysfunction/failure in intensive care units.

Our patient had a history of multiple risk factors including her age, hypertension, and history of heart failure with an implantable pacemaker and chronic lower extremity edema. The infection from her skin breakdown in her lower extremity most likely caused cellulitis, bacteremia, and TSS. She survived up to 46 days post admission perhaps due to timely initiation of IVIG as an adjunctive treatment in addition to antibiotics. 

## Conclusions

TSS from SDSE can cause serious life-threatening complications including multi-organ failure and death. SDSE is more prominent in elderly patients with preexisting co-morbidities. Our patient did not show clinical improvement despite initiation of multiple vasopressors and broad spectrum antibiotics which raised suspicion for TSS. IVIG was initiated as an adjunctive treatment with antibiotics. One could only speculate that the use of IVIG prolonged our patients survival. We therefore emphasize timely diagnosis and treatment with antibiotics, IVIG, and surgical consult to rule out necrotizing fasciitis in suitable patients to help prevent progressive disease and mortality. 
